# Posterior Reversible Encephalopathy Syndrome Resolving Within 48 Hours in a Normotensive Patient Who Underwent Thoracic Spine Surgery

**DOI:** 10.14740/jocmr2472w

**Published:** 2016-01-26

**Authors:** Kunal Vakharia, Ioannis Siasios, Vassilios G. Dimopoulos, John Pollina

**Affiliations:** a Department of Neurosurgery, Jacobs School of Medicine and Biomedical Sciences, University at Buffalo, State University of New York, Buffalo, NY, USA; b Department of Neurosurgery, Kaleida Health, Buffalo, NY, USA

**Keywords:** Prone surgery, Posterior reversible encephalopathy syndrome, Cortical blindness

## Abstract

Posterior reversible encephalopathy syndrome (PRES) usually manifests with severe headaches, seizures, and visual disturbances due to uncontrollable hypertension. A patient (age in the early 60s) with a history of renal cell cancer presented with lower-extremity weakness and paresthesias. Magnetic resonance imaging (MRI) of the thoracic spine revealed a T8 vertebral body metastatic lesion with cord compression at that level. The patient underwent preoperative embolization of the tumor followed by posterior resection and placement of percutaneous pedicle screws and rods. Postoperatively, the patient experienced decreased visual acuity bilaterally. Abnormal MRI findings consisted of T2 hyperintense lesions and fluid-attenuated inversion recovery changes in both occipital lobes, consistent with the unique brain imaging pattern associated with PRES. The patient’s blood pressure was normal and stable from the first day of hospitalization. The patient was kept on high-dose steroid therapy, which was started intraoperatively, and improved within 48 hours after symptom onset.

## Introduction

Perioperative visual loss associated with spine surgery has been extensively described [1-3]. Numerous causes could provoke this pathological clinical entity, depending on the anatomical topography of the spinal lesion. Vision loss occurring in conjunction with spine surgery may result from anterior or posterior ischemic optic neuropathy, central retinal artery occlusion, cortical blindness, and posterior reversible encephalopathy (PRES) [4]. Practice advisories regarding perioperative visual loss associated with spine surgery were published by the American Society of Anesthesiologists in an effort to improve knowledge of the diagnosis and treatment of this clinical finding [5, 6]. The reported incidence of perioperative visual loss in spine fusions is 3.09/10,000 [7].

As mentioned, one cause of this perioperative loss of vision is PRES. First described by Hinchey et al [8], PRES is a rare entity typically associated with severe acute hypertension, eclampsia, renal failure, electrolyte disorders, hematologic disorders, infections, immunosuppressive therapy, chemotherapy, and transfusions [9-18]. The main clinical findings are headaches, visual impairment, and neurological disorders. The diagnosis is established with brain magnetic resonance imaging (MRI) that reveals characteristic high signal lesions on T2-weighted images and fluid-attenuated inversion recovery (FLAIR) sequences in both occipital lobes.

Here we describe the rare case of a normotensive patient who developed PRES after removal of a spinal metastasis of renal cell origin that was located at the T8 vertebrae.

## Case Report

A patient (age in the early 60s) presented with back pain, sensory deficit from the level of T8 and below and progressive weakness in both lower extremities. On examination at the time of the current admission, the patient was noted to have urinary retention and diminished rectal tone. The patient underwent a nephrectomy 3 years prior for renal cell carcinoma and had never undergone chemotherapy or radiation therapy after the resection of her primary tumor. There was no medical history of stroke, heart disease, or hypertension.

MRI of the thoracic spine revealed a large metastatic lesion in the T8 vertebral body that compressed the spinal cord at that level. It was decided first to cut off the blood supply to this vertebra metastatic lesion by embolization. This could minimize blood loss before surgical decompression of the tumor.

After informed consent was obtained, the embolization was accomplished under general anesthesia in the angiosuite, and the patient was placed on high-dose steroid therapy (dexamethasone) in an effort to reduce subsequent post-surgical edema. The patient was then taken to the operating room and placed in a prone position. After resection of the tumor and percutaneous placement of pedicle screws and rods at T7 and T9, the patient was taken to the neurointensive care unit for close monitoring. Blood pressure readings were recorded meticulously, and the patient was never hypertensive or hypotensive (Table 1).

**Table 1:** Hematocrit/Hemoglobin, Platelet Count, and Serum Creatinine Urea Values From Initial Hospitalization Through Discharge From the NeuroICU

	Before surgery	During surgery	NeuroICU
Blood pressure (mm Hg)	124/62	130/67, 100/64, 148/81	Range: 140/65 - 150/70
Hematocrit (%)/hemoglobin (mg/dL)	40/13.6	38/13.6	31/9.8
Platelet count (× 10^3^/mL)	226	170	288
Serum creatinine/urea (mg/dL)	0.98/18	0.76/9	0.74/19

NeuroICU: neurointensive care unit.

In the first hours after surgery, the patient experienced decreased visual acuity bilaterally to nearly 20/400 OU. Although baseline visual acuity was not recorded preoperatively, no visual problems were experienced in the past, according to the patient and family. The patient denied any pain, double vision, or other neurological symptoms. Both eyes were normal in appearance, without any sign of external injury.

Given the unique presentation, cardiologists, neurologists, neurosurgeons, and ophthalmologists were involved in attempting to determine the diagnosis. Although the initial picture suggested ischemic neuropathy, there were no funduscopic changes on the ophthalmologic examination indicative of central retinal artery occlusion or ischemic infarction.

**Figure 1:**
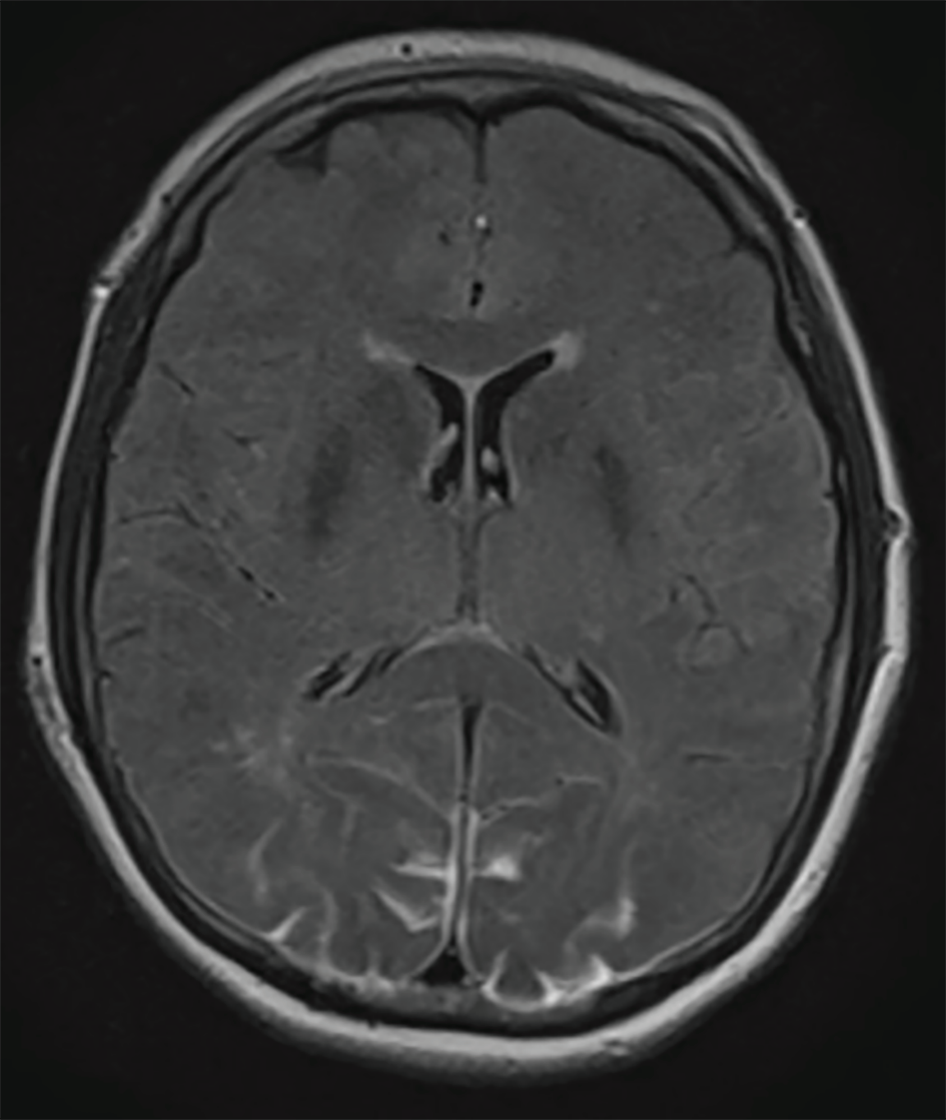
Axial FLAIR MRI demonstrating bilateral occipital lobe hyperintensity, suggestive of blood or edema. Because the images from other brain MRI sequences were normal, the patient was diagnosed with PRES.

An urgent MRI of the orbits and brain was performed. There were no pathological findings in the orbits. Brain MRI showed occipital lesions with T2 hyperintensity and pathological signal in FLAIR sequence in subcortical regions of both occipital lobes, suggestive of PRES (Fig. 1). A postoperative stroke study showed no signs of decreased perfusion.

**Figure 2:**
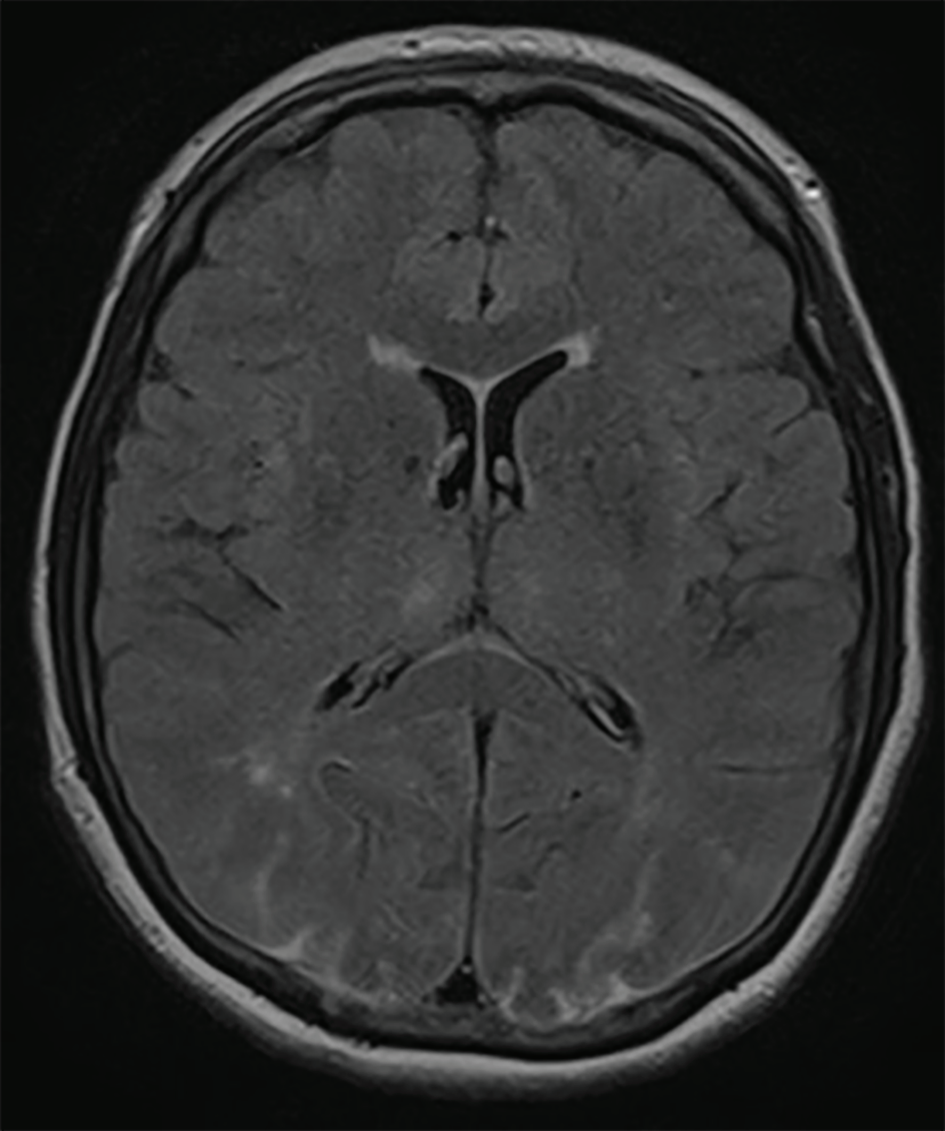
Axial FLAIR MRI demonstrating improvement in the hyperintense lesions in both occipital lobes 48 h after symptom onset.

The patient remained on high-dose steroid therapy postoperatively after ophthalmologist’s consultation who recommended 4 days of high-dose steroids (dexamethasone) and then taper over 48 h with a dose of 4 mg twice a day. Within 48 h after symptom onset, visual acuity improved according to ophthalmologist’s re-evaluation to 20/200. Brain MRI findings were also improved within 48 h (Fig. 2). At the last follow-up 2 months after surgery, the patient reported no visual disturbances.

## Discussion

According to Bartynski [19, 20], the development of PRES can be explained on the basis of two theories, although the exact mechanism is still unknown. These theories are 1) severe hypertension that leads to failed autoregulation, hyperperfusion, and finally endothelial injury/vasogenic edema; and 2) vasoconstriction and hypoperfusion that lead to brain ischemia and subsequent vasogenic edema. In our case, there were no hypertensive episodes during surgery or immediately postoperatively so our case is a normotensive PRES case, which has been described only in non-spine surgery cases, as documented by Bartynski [20]. In their retrospective study, de Havenon et al [21] reported two cases of PRES in patients who presented with loss of visual acuity without having spine surgery. Imaging studies revealed the typical T2/FLAIR hypertensive lesions of the brain in addition to spinal cord involvement. These two patients had acute onset of hypertension and treated with the administration of antihypertensive drugs. One of these patients also received steroid therapy (methylprednisolone for 3 days).

Intraoperative hypotension and insufficient blood flow can lead to cortical blindness after ocular or non-ocular surgical procedures, as Berg et al [22] claimed in their study. Our patient was normotensive during the two-stage procedure and postoperatively, as vital sign monitoring proved. In the recent literature, five cases of PRES related to spine surgery were reported [23-27]. According to the authors of these cases, the possible pathophysiologic mechanisms were related to hypotension [23], severe anemia [24], prone positioning [25], hypertension due to an epidural thoracic test with bupivacaine [26], and autonomic dysreflexia in a cervical trauma case with spinal cord involvement [27]. Prone positioning during spine surgery could lead to PRES from the increase in cerebrospinal fluid pressure [3, 25]. Another cause for PRES is embolism, which is a very uncommon phenomenon if it is present in bilateral cortical areas and is more frequent in cardiovascular anomalies [28, 29]. There are some studies reporting PRES in patients with renal failure [16], hematological disorders [12, 13], and transfusion [18]. Our patient did not receive a transfusion, and the results of laboratory tests for renal function, hematocrit, platelets, and red blood cells were normal. She did receive high-dose steroid therapy before surgery, which has been implicated in the genesis of PRES in a few non-spine studies [30-32]. None of the described spine surgery cases received a course of high-dose steroids before the procedure.

Most patients with PRES are treated for their hypertension, and some have benefitted from postoperative steroid therapy [33, 34]. The present case demonstrates a novel appearance of normotensive PRES that resolved with continued high-dose steroids within 48 h. In most patients with PRES, MRI studies do not show significant improvement within 24 - 48 h of onset [21]. In our patient, significant changes were seen on MRI obtained 48 h after the initial clinical signs. To our knowledge, that has not been documented before. Anesthesiologists and spine surgeons should be aware of this clinical syndrome as a reason for transient and reversible vision loss in the setting of prone spine surgery.

### Conclusion

PRES is an uncommon clinical entity in spine surgery. PRES in relation to normotension after prone spine surgery is an event that has not been reported thus far in the literature. Documentation of pathological lesions located in the bilateral occipital lobes that are seen on FLAIR-T2 MRI and improve on MRI obtained within 48 h after surgery is unique in the literature. Spine surgeons and anesthesiologists should be aware of the clinical and imaging characteristic of this syndrome.

## Abbreviations

CT: computed tomography
FLAIR: fluid-attenuated inversion recovery
MRI: magnetic resonance imaging
PRES: posterior reversible encephalopathy syndrome
